# Periodically stimulated piecewise linear adaptive exponential integrate-and-fire neuron

**DOI:** 10.1186/1471-2202-14-S1-P234

**Published:** 2013-07-08

**Authors:** LieJune Shiau, Carlo Laing

**Affiliations:** 1 Department of Mathematics, University of Houston, Clear Lake, Houston, TX, USA; 2 Institute of Information and Mathematical Sciences, Massey University, Auckland, New Zealand

## 

Although variability is a ubiquitous characteristic of the nervous system, under appropriate conditions neurons can generate precisely timed action potentials. Thus considerable attention has been given to the study of a neuron's output in relation to its stimulus. Frequency selectivity of mode locking exhibits in clinical studies of human cardiorespiratory systems [1], experimental sensory processing in the auditory nerve [2] and hair cells in cochlea [3], and experimental brain study in thalamocortical relay neuron response [4]. To understand the mechanisms behind these functional features, spiking neuron models are used in studying the precise timing of firing events that is thought to underlie the frequency mode locking. In this study, we consider an increasingly popular model, the adaptive exponential integrate- and-fire (aEIF) neuron, and, for analytical tractability, its piecewise linear variant is adopted to understand the responses of such neurons to periodic stimuli.

**Figure 1:**
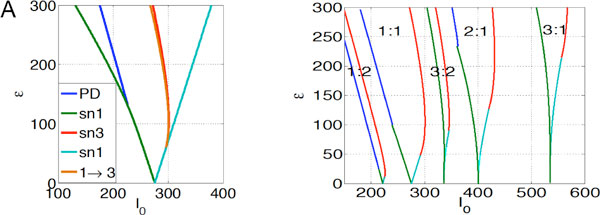
**(**A**) Various bifurcations shown in 1:1 phase locked solutions**. PD: Period-doubling; sn1: saddle-node in switching manifold once; sn3: saddle-node in switching manifold three times, 1 → 3, transition from sn1 to sn3. (**B**) Frequency mode locked solutions shown in the marked Arnol'd tongue structure.

We construct general solutions of arbitrary mode locked states driven by a sinusoidal external stimulus, and describe their stability by the maximal Lyapunov exponent. We find period-doubling and saddle-node bifurcations of mode locked solutions, and the instabilities of the mode locked states lead to a so-called Arnol'd tongue structure in parameter space (Figure 1 shown in input amplitude versus current), with p:q denoting the neuron firing p times for every q periods of the stimuli (Figure 1B). An interesting aspect of this model that is unaware of occurring elsewhere, is that if the amplitude of periodic stimulus is sufficiently large, solutions can enter different regions of phase space (switching manifold) multiple times between firing. Significant parts of the boundaries of Arnol'd tongues are defined by saddle-node bifurcations of such solutions (marked as red in Figure 1).

The aEIF model includes a subthreshold and a spike-triggered adaptation parameters which can be related to the M-current and the afterhyperpolarization current, respectively, both producing spike-frequency adaptation. We observe similar tongue structures when either adaptation value is increased. It consists with the finding shown [5] that higher values of input current are required to maintain the same spiking frequency with an increase of either adaptation parameter. The theoretical analysis is in excellent agreement with numerical simulations, and this study can be used to further understand the functional features related to responses of such neurons to biologically realistic stimuli.
